# An educational intervention improved knowledge of dietary supplements in college students

**DOI:** 10.1186/s12889-020-08786-3

**Published:** 2020-05-07

**Authors:** Tsuyoshi Chiba, Etsuko Kobayashi, Takashi Okura, Masashi Sekimoto, Hideya Mizuno, Maki Saito, Keizo Umegaki

**Affiliations:** 1 Department of Food Function and Labeling, National Institute of Health and Nutrition, National Institutes of Biomedical Innovation, Health and Nutrition, 1-23-1 Toyama, Shinjuku-ku, Tokyo, 162-8636 Japan; 2 Laboratory of Pharmaceutics, Faculty of Pharma-Sciences, Teikyo University, Tokyo, Japan; 3 Laboratory of Environmental Hygiene, Department of Environmental Science, School of Life and Environmental Science, Azabu University, Kanagawa, Japan; 4 School of Pharmacy and Pharmaceutical Sciences, Mukogawa Women’s University, Hyogo, Japan; 5 Division of Molecular and Cellular Pharmacology, Department of Pathophysiology and Pharmacology, School of Pharmacy, Iwate Medical University, Iwate, Japan; 6 Department of Food Safety and Management, Showa Women’s University, Tokyo, Japan

**Keywords:** Dietary supplements, College students, Education, Pharmacists

## Abstract

**Background:**

We have previously reported on the prevalence of dietary supplements among college students; it was deduced that their intake of supplements increased according to their grade (i.e., 13.1% in the first grade to 20.5% in the sixth grade). We also reported that some students had experienced adverse events in Japan due to their intake of these supplements. However, awareness of dietary supplements among college students remains limited, even among pharmaceutical students. Being appropriately educated about them is important for pharmaceutical students, both for themselves as well as for their future careers as pharmacists.

**Methods:**

We conducted a lecture-based educational intervention about dietary supplements on 328 college students in Japan—184 from pharmaceutical science and 144 from environmental science or food and life science disciplines. The purpose of this study was to evaluate the effects of an educational intervention on college students’ understanding of dietary supplements. The intervention involved a lecture that covered the quality of dietary supplements, how they differed from drugs, and a summary of their adverse events. The lecture was evaluated using a 14-question questionnaire. We then compared the pre- and post-intervention responses to the same questionnaire using a Wilcoxon signed-rank test. The questions were assessed using a Likert scale that ranged from “strongly agree” to “strongly disagree”; the latter being the preferred answer.

**Results:**

Before the intervention had taken place, the students’ understanding of dietary supplements was shown to be deficient. Conversely, post-intervention, their knowledge levels had significantly improved, especially concerning agreement on whether “Dietary supplements are safe because they are just food items”. Pre-intervention, 2.7% strongly agreed and 37.5% agreed; post-intervention, 1.2% strongly agreed and 15.6% agreed. On whether “Dietary supplements made from natural ingredients or herbs are safe”, at the pre-intervention stage 2.8% strongly agreed and 44.0% agreed and post-intervention, 2.2% strongly agreed and 16.9% agreed. On whether “Dietary supplements made from food items are safe”, 4.0% strongly agreed and 43.6% agreed pre-intervention and 0.9% strongly agreed and 16.6% agreed post-intervention. Despite there being a greater number of pharmaceutical students who had a correct understanding of dietary supplements before the intervention, these students still showed improvement after the lecture.

**Conclusion:**

An intervention in the form of a single educational lecture has the capacity to improve college students’ understanding of dietary supplements. It is important for pharmacists to be appropriately educated about dietary supplements when they consult with patients. We will evaluate the long-term effects of the intervention on the alumni (pharmacists) in a subsequent study.

## Background

Dietary supplements, which include vitamins, minerals, and herbal supplements, are widely used across multiple generations. However, in previous studies, we reported various inappropriate uses of dietary supplements. Many mothers believe that they are suitable for children that are picky eaters [1], which is in line with a study that showed that an unfavorable diet in children may be accompanied by dietary supplements [2]. In college students, males predominantly used protein supplements for building muscle, whereas females favored dietary supplements for weight loss [3]. Our study found that many pregnant women used dietary supplements without exact knowledge of appropriate intake timing and were generally unaware of product safety [4]. We also found that many patients used dietary supplements to treat their diseases without consulting with their doctors [5]. Adverse events associated with dietary supplements occurred in each generation [1, 3, 5, 6].

In general, adverse events such as diarrhea, vomiting, and liver injury have reportedly been associated with the use of herbal supplements [7–9]. Adverse events may either occur because there is an issue with the product or with how consumers consume them. Concerning the product itself**,** adverse events may occur when the product has been contaminated with large amounts of heavy metals, undeclared allergens, or are adulterated with illegal drug substances. In these cases, adverse events can lead to serious consequences [6, 10]. However, government ministries and regulatory agencies routinely conduct market surveillance checks to eliminate these sorts of risks, so adverse events caused by these types of products are rare in Japan [11]. Concerning the consumers’ use of the product, adverse events may also occur due to inappropriate use. For example, they may occur through the consumption of an excessive amount, concomitant use of multiple dietary supplements, or concomitant use with medicines.

Although a positive association between the use of dietary supplements and education levels has been reported [12–14], several studies have indicated that consumers’ knowledge of dietary supplements is limited among older adults and patients [15, 16]. A survey of the elderly in the United States revealed that 19% of current dietary supplement users were taking supplements with medicines in potentially harmful combinations [15]. To prevent adverse events through the inappropriate use of dietary supplements, further education appears necessary.

We have previously conducted a nationwide internet survey of the prevalence of dietary supplement use among college students in Japan [3]. The prevalence of their use increased according to the students’ grades (from 13.1% in the first grade to 20.5% in the sixth grade) and it was higher in medical and pharmaceutical college students (22.0%) than in others (16.7%). College students, especially pharmaceutical students, learn about food, nutrition, and dietary supplements in their courses at university. Some students take dietary supplements to obtain adequate amounts of nutrients and maintain their health. However, others use dietary supplements for weight loss and the prevention of and treatment of diseases and have experienced adverse events. These results suggest that the current state of education at pharmaceutical colleges in Japan is inadequate in ensuring that dietary supplements are consumed appropriately. In addition, several studies have been conducted that focus on the extent of the knowledge that university or college students have of dietary supplements [17–19]. One Serbian study suggested that medical students educated in pharmacology were likely to have a better understanding of the risks of dietary supplements than their counterparts [18]. On the other hand, a study from the United States concluded that knowledge concerning dietary supplements was limited even among pharmaceutical college students [17]. Their knowledge about health benefit claims or the adverse reactions of each ingredient was also limited. They also did not know that the FDA did not require dietary supplements to be proven to be safe and effective before marketing and that it monitored the safety of dietary supplements only after they were on the market. Limited opportunities for further education on the topic might be the cause behind this lack of knowledge. These differences may be due to what students had learned in college about dietary supplements, where it appears that more information is provided on corresponding laws and institutions rather than on their appropriate use. Therefore, it is necessary for college students to receive further education that focuses on the safety and health risks involved with dietary supplements.

It is not clear what the level of knowledge concerning dietary supplements is among college students in Japan, because the quality of lectures given on dietary supplements might depend on the respective departments or colleges. In addition, there has as yet been no study that identifies the efficacy of regular lectures on students’ knowledge of dietary supplements. In this study, we aimed to clarify what is currently known about dietary supplements among college students—especially those studying pharmaceutical science—and to evaluate the effects of a lecture-based educational intervention on their understanding.

## Methods

### Study design and participants

An educational intervention was undertaken between June and December 2017 wherein questionnaires were completed by college students before and after attending a lecture. The participants comprised of 328 college students from Teikyo University, Mukogawa Women’s University, Iwate Medical University, and Azabu University; 184 students from pharmaceutical sciences (Teikyo University, Mukogawa Women’s University, and Iwate Medical University); 66 students from environmental sciences; and 78 students from food and life sciences (Azabu University). The pharmaceutical sciences students had learned about the laws and institutions related to dietary supplements in regular lectures prior to the intervention lecture, while students from environmental sciences and food and life sciences had not. To assess the effects of the educational intervention on the students’ understanding of dietary supplements, we used an assessment scale comprising of 14 questions. Students answered the questionnaire just before the lecture (pre-intervention) and immediately after the lecture (post-intervention). The changes in their answers were evaluated.

### Educational intervention

An educational intervention was conducted in the form of a once-off one-hour lecture. The lecture was comprised of information concerning the safety of dietary supplement use, the quality of dietary supplements, the possibility of interactions between dietary supplements and medicines, and adverse events that may occur due to their use. The first author delivered the same lecture at all four colleges. Educational interventions were conducted during a regular class period at Mukogawa Women’s University, Iwate Medical University, and Azabu University. Students in the classes where the lecture was being offered were, therefore, compelled to attend. At Teikyo University, the intervention was conducted as a special lecture. The date, place, and a summary of the lecture contents were announced at other lectures and students could voluntarily choose whether or not to attend. In addition, all of the students attending the lecture could choose whether or not to complete the questionnaire without incurring any penalty if they did not.

### Questionnaire

We used a self-administered and paper-based questionnaire. The survey sheet was separated into pre- and post-intervention parts. The pre-intervention part included demographic characteristics (age and sex), the prevalence of dietary supplement use, and questions that determined their understanding of dietary supplements. A dietary supplement is not defined by law in Japan and some dairy and soybean products are considered to be dietary supplements even though they are in the form of common food items. Therefore, the meaning of the term “dietary supplement” is likely to differ among individuals. In this survey, a “dietary supplement” was defined to be what college students considered to have beneficial effects on their health (e.g., vitamins, minerals, fish oil, amino acids, dietary fiber, probiotics, and energy drinks). In addition, current users were asked about their sources of information concerning these supplements, how such products could be obtained, the purpose of dietary supplement use, and the number of dietary supplements they used. We prepared 14 questions on the students’ knowledge of dietary supplements based on the regulation in Japan and on the inappropriate knowledge of their use in Japanese that was sourced from previous studies [1–5, 20–23]. The 14 questions (including one control question) were assessed using a 5-point Likert scale ranging from “strongly agree” to “strongly disagree”. All of the questions were designed to have “strongly disagree” as the most preferable answer (Supplemental Table). For example, supplement providers generally encourage consumers to purchase their products by exploiting ideas such as “Dietary supplements are safe because they are just food items” or “Dietary supplements made from natural ingredients or herbs are safe” even though they do not manage the safety of their products. In this regard, answering “strongly disagree” to those questions demonstrates the preferable viewpoint. In the same manner, the most preferable answers to the other questions were set at “strongly disagree” with the current situation of Japan in mind. Post-intervention, the students answered the same 14 questions as they had in the pre-intervention part of the study.

### Statistical analyses

Chi-squared tests were used to assess significant differences between the sexes. To evaluate the educational effects of the intervention on students’ understandings, the pre- and post-intervention differences were assessed as a z-score with the Wilcoxon signed-rank test. Students’ understandings of dietary supplements were compared between current supplement users and non-users and between pharmaceutical science students and other department students (environmental science and food and life science) using the Mann-Whitney U test. All statistical analyses were performed with Stata/IC 15 (Light Stone, Tokyo, Japan) and a *p*-value < 0.05 was considered statistically significant.

## Results

### Students characteristics

Three hundred fifty-two students at four colleges (Azabu University, Iwate Medical University, Mukogawa Women’s University, and Teikyo University) attended the intervention. Of these, 328 students (150 males and 178 females) completed both pre- and post-intervention questionnaires (response rate of 93.2%) and the data were subsequently analyzed. The age of the students ranged from 18 to 38 years old (mean ± SD; 21.9 ± 2.5). More than half of the students (184) belonged to pharmaceutical science departments.

### The prevalence of dietary supplement use

The prevalence of dietary supplement use is shown in Table 1. Overall, 85 students (25.9%) were using one or more dietary supplements at the time the questionnaire was administered. Ninety-six students (29.3%) had previously used dietary supplements and almost half of the students (44.8%) had never used them. The prevalence of any type of dietary supplement (vitamin/mineral and/or non-vitamin/mineral supplement) use was slightly higher in females (29.2%) than in males (22.0%), but this was not statistically significant. Among the current users (*n* = 85), 66 students (77.6%) were only using vitamin/mineral supplements, seven students (8.2%) were only using non-vitamin/mineral supplements, and 12 students (14.1%) were using both vitamin/mineral and non-vitamin/mineral supplements. More than half of the current users (56.5%) were using more than two dietary supplements at the same time. The prevalence was higher in pharmaceutical students (28.8%) than in other students (22.2%), but this difference was not statistically significant.

**Table 1:** Prevalence of dietary supplement use

	n	Currently Using			Previously Used	Never Used
		Any type	Vitamin Mineral	Non-Vitamin Non-Mineral		
All (%)	328	25.9	22.3	5.8	29.3	44.8
**Sex**						
Males	150	22.0	18.0	5.3	28.7	49.3
Females	178	29.2	25.8	6.2	29.8	41.0
**Department**						
Pharmaceutical	184	28.8	25.0	7.1	32.1	39.1
Others	144	22.2	18.8	4.2	25.7	52.1

Statistical analyses were conducted between male and female students and between pharmaceutical students and other students

### Information sources on dietary supplements and how they obtained dietary supplements

We asked students who were using or had previously used dietary supplements about the sources they consult to obtain information on what they consume (Table 2). Most of the students in this study got information on dietary supplements from the internet (40.0% in all) followed by their families (35.3%) and in-store advertisements (27.1%). Females predominantly gained information through in-store advertisements. The way how they obtained dietary supplements is shown in Table 3. Although most students bought their dietary supplements themselves (60.0% in all), more than half of the students obtained dietary supplements via their family members (50.6%). No gender differences were observed.

**Table 2:** Sources of information about dietary supplements used by college students

	All (85)	Males (33)	Females (52)	*p*-value
Internet (%)	40.0	30.3	46.2	0.109
Family	35.3	30.3	38.5	0.368
In-store advertisements	27.1	15.2	34.6	0.038
Product labels	25.9	18.2	30.8	0.163
Television	22.4	15.2	26.9	0.173
Pharmacists or drug store clerks	21.2	21.2	21.2	0.932
Friends or acquaintances	10.6	9.1	11.5	0.677
Clinic (physicians, pharmacists, dietitians)	9.4	3.0	13.5	0.097
Newspapers, magazines, flyers	8.2	6.1	9.6	0.390
Mentors	3.5	0.0	5.8	0.152
Coaches	3.5	6.1	1.9	0.332
Others	2.4	3.0	1.9	0.765

Multiple answers. Statistical analyses were conducted between male and female students

**Table 3:** How college students obtained dietary supplements

	All (85)	Males (33)	Females (52)	*p*-value
By myself (%)	60.0	60.6	59.6	0.898
Family	50.6	42.4	55.8	0.165
College or club that encourages use	2.4	3.0	1.9	0.414

Multiple answers. Statistical analyses were conducted between male and female students

### The purposes of dietary supplement use

We also asked students who were using or had previously used dietary supplements about the purposes behind their use (Table 4). Most students were using them to supplement their nutrient intake (70.6% in all) and to maintain their health (42.4%). Some students were using supplements to prevent (16.5%) or treat (8.2%) diseases. There were no significant gender differences with regard to this point. However, taking dietary supplements to build muscle and progress athletic performance occurred at three times the rate in males compared to females.

**Table 4:** Purpose of dietary supplement use

	All (85)	Males (33)	Females (52)	*p*-value
Supplementation of nutrients (%)	70.6	69.7	71.2	0.668
Maintenance of health	42.4	45.5	40.4	0.756
Improvements to health	30.6	27.3	32.7	0.518
Prevention of diseases	16.5	15.2	17.3	0.734
Progress academic performance	11.8	9.1	13.5	0.501
Building muscle	10.6	18.2	5.8	0.081
Weight loss	8.2	6.1	9.6	0.527
Treatment of diseases	8.2	6.1	9.6	0.527
For growth	5.9	6.1	5.8	0.991
Progress athletic performance	3.5	6.1	1.9	0.332
Others	7.1	3.0	9.6	0.230

Multiple answers. Statistical analyses were conducted between male and female students

### The effects of the educational intervention on the students’ understanding of dietary supplements

We assessed the students’ understanding of dietary supplements using a 14-question questionnaire (Table 5). In the pre-intervention questionnaire, 47.6% of students had an incorrect understanding in relation to “Dietary supplements made from food items are safe” (4.0% of which strongly agreed and 43.6% agreed), whereas the proportion of students who agreed with this question was reduced to 17.5% (0.9 and 16.6%, respectively) in the post-intervention questionnaire (z = − 10.700, *p* < 0.01). Similarly, the proportion of students who agreed with “Dietary supplements made from natural ingredients or herbs are safe” was reduced from 46.8% (2.8 and 44.0%) to 19.1% (2.2 and 16.9%) (z = − 10.280, p < 0.01). Regarding the efficacy of dietary supplements, 27.7% (0.9 and 26.8%) of students believed that “Dietary supplements can prevent diseases” and 25.9% (1.8 and 24.1%) of students believed that “Dietary supplements can compensate for an unbalanced diet” before the intervention. These opinions changed after the intervention to 17.8% (1.5 and 16.3%) and 15.5% (0.6 and 14.9%), respectively. It was found that post-intervention, understandings of dietary supplements were significantly changed from the pre-intervention phase in all questions apart from “Food additives should be avoided” (z = 1.836, *p* = 0.07). Information on food additives was not mentioned in the lecture because it was a control question.

**Table 5:** Comparison of knowledge about dietary supplements between pre- and post-intervention

		Strongly agree (%)	Agree (%)	Neither agree nor disagree (%)	Disagree (%)	Strongly disagree (%)	Z score	*p*-value
Dietary supplements are safe because they are just food items.	Pre	2.7	37.5	25.3	30.2	4.3	−8.497	< 0.01
	Post	1.2	15.6	23.6	47.9	11.7		
Dietary supplements made from natural ingredients or herbs are safe.	Pre	2.8	44.0	28.4	21.1	3.7	−10.280	< 0.01
	Post	2.2	16.9	28.2	42.9	9.8		
Food additives should be avoided.	Pre	3.4	36.6	29.9	26.5	3.7	1.836	0.07
	Post	5.9	35.8	30.9	23.5	4.0		
Dietary supplements made from food items are safe.	Pre	4.0	43.6	29.5	21.2	1.8	−10.700	< 0.01
	Post	0.9	16.6	28.2	43.3	11.0		
The efficacy of commercial dietary supplements is confirmed and reliable.	Pre	2.1	18.7	33.6	36.4	9.2	−8.102	< 0.01
	Post	0.9	9.5	18.1	46.6	24.9		
I want to use dietary supplements that have a good reputation.	Pre	3.4	31.7	18.9	32.6	13.4	−9.959	< 0.01
	Post	1.5	8.6	17.9	44.3	27.7		
Dietary supplements recommended by health professionals are effective.	Pre	0.9	15.9	22.6	38.5	22.0	−7.382	< 0.01
	Post	0.9	5.5	12.9	48.2	32.5		
Dietary supplements can be used concomitantly with medicines.	Pre	1.2	8.9	15.0	44.0	30.9	−7.215	< 0.01
	Post	0.3	2.8	9.5	36.7	50.8		
Dietary supplements can prevent diseases.	Pre	0.9	26.8	23.8	36.3	12.2	−6.300	< 0.01
	Post	1.5	16.3	16.0	41.7	24.5		
Dietary supplements can treat diseases.	Pre	0.3	6.4	18.4	40.1	34.9	−4.855	< 0.01
	Post	0.0	3.1	11.3	40.1	45.6		
Dietary supplements can compensate for an unbalanced diet.	Pre	1.8	24.1	20.7	38.7	14.6	−5.168	< 0.01
	Post	0.9	13.6	20.4	41.4	23.8		
Children who are picky eaters should take dietary supplements to supplement nutrition.	Pre	0.6	14.9	15.2	43.0	26.2	−4.440	< 0.01
	Post	0.3	8.9	15.0	40.4	35.5		
Pregnant women should take dietary supplements to supplement nutrition.	Pre	2.5	20.6	24.5	37.7	14.7	−8.003	< 0.01
	Post	1.2	12.6	18.7	38.7	28.8		
I want to use dietary supplements for weight loss or muscle building.	Pre	4.0	22.3	17.1	32.6	24.1	−6.342	< 0.01
	Post	3.1	12.6	13.2	36.5	34.7		

Pre: pre-intervention; Post: post-intervention

Statistics analyses were conducted between the pre- and post-intervention answers to the questionnaire

### Subgroup analysis

We compared the understandings of dietary supplements between users (those currently using supplements) and non-users (those who had previously used supplements or had never used them) (Table 6). At the pre-intervention stage, users were more likely to agree with the following: “The efficacy of commercial dietary supplements is confirmed and reliable” (z = 2.203, *p* = 0.03), “Dietary supplements recommended by health professionals are effective” (z = 2.579, *p* = 0.01), “Dietary supplements can prevent diseases” (z = 4.674, *p* < 0.01), “Dietary supplements can treat diseases” (z = 2.051, *p* = 0.04), “Dietary supplements can compensate for an unbalanced diet” (z = 5.269, *p* < 0.01), and “Children who are picky eaters should take dietary supplements to supplement nutrition” (z = 2.221, *p* = 0.03). There remained differences between users and non-users in relation to two questions, namely “Dietary supplements can prevent diseases” (z = 2.351, *p* = 0.02) and “Dietary supplements can compensate for an unbalanced diet” (z = 3.075, *p* < 0.01) at the post-intervention stage. However, the understandings of these questions had improved among both users and non-users.

**Table 6:** Comparison of knowledge about dietary supplements between current users and former or never users

		Pre-intervention							Post-intervention							
		Strongly agree (%)	Agree (%)	Neither agree nor disagree (%)	Disagree (%)	Strongly disagree (%)	Z score	*p*-value^a^	Strongly agree (%)	Agree (%)	Neither agree nor disagree (%)	Disagree (%)	Strongly disagree (%)	Z score	*p*-value^a^	*p*-value^b^
Dietary supplements are safe because they are just food items.	Users	5.9	41.2	20.0	31.8	1.2	1.451	0.15	2.4	15.3	21.2	47.1	14.1	−0.429	0.67	< 0.01
	Non-users	1.7	36.2	27.2	29.6	5.4			0.8	15.8	24.5	48.1	10.8			< 0.01
Dietary supplements made from natural ingredients or herbs are safe.	Users	3.5	50.6	23.5	21.2	1.2	1.478	0.14	3.5	16.5	25.9	44.7	9.4	−0.009	0.99	< 0.01
	Non-users	2.5	41.7	30.2	21.1	4.6			1.7	17.0	29.0	42.3	10.0			< 0.01
Food additives should be avoided.	Users	1.2	35.3	28.2	29.4	5.9	−1.310	0.19	4.8	36.9	27.4	25.0	6.0	−0.556	0.58	0.10
	Non-users	4.1	37.0	30.5	25.5	2.9			6.3	35.4	32.1	22.9	3.3			0.26
Dietary supplements made from food items are safe.	Users	8.2	42.4	31.8	17.7	0.0	1.456	0.15	2.4	17.6	28.2	42.4	9.4	0.800	0.42	< 0.01
	Non-users	2.5	44.0	28.6	22.4	2.5			0.4	16.2	28.2	43.6	11.6			< 0.01
The efficacy of commercial dietary supplements is confirmed and reliable.	Users	3.6	21.4	39.3	31.0	4.8	2.203	0.03	2.4	6.0	20.2	48.8	22.6	0.235	0.81	< 0.01
	Non-users	1.7	17.7	31.7	38.3	10.7			0.4	10.7	17.4	45.9	25.6			< 0.01
I want to use dietary supplements that have a good reputation.	Users	7.1	35.3	20.0	25.9	11.8	1.941	0.05	2.4	14.1	18.8	42.4	22.4	1.998	0.05	< 0.01
	Non-users	2.1	30.5	18.5	35.0	14.0			1.3	6.7	17.5	45.0	29.6			< 0.01
Dietary supplements recommended by health professionals are effective.	Users	0.0	23.5	28.2	31.8	16.5	2.579	0.01	0.0	7.1	15.5	48.8	28.6	1.049	0.29	< 0.01
	Non-users	1.2	13.2	20.7	40.9	24.0			1.2	5.0	12.0	47.9	33.9			< 0.01
Dietary supplements can be used concomitantly with medicines.	Users	3.5	9.4	21.2	38.8	27.1	1.800	0.07	1.2	3.5	8.2	36.5	50.6	0.116	0.91	< 0.01
	Non-users	0.4	8.7	12.8	45.9	32.2			0.0	2.5	9.9	36.8	50.8			< 0.01
Dietary supplements can prevent diseases.	Users	1.2	43.5	24.7	28.2	2.4	4.674	< 0.01	1.2	27.1	14.1	38.8	18.8	2.351	0.02	< 0.01
	Non-users	0.8	21.0	23.5	39.1	15.6			1.7	12.4	16.6	42.7	26.6			< 0.01
Dietary supplements can treat diseases.	Users	0.0	10.7	22.6	38.1	28.6	2.051	0.04	0.0	5.9	10.6	42.4	41.2	1.060	0.29	< 0.01
	Non-users	0.4	4.9	16.9	40.7	37.0			0.0	2.1	11.6	39.3	47.1			< 0.01
Dietary supplements can compensate for an unbalanced diet.	Users	3.5	45.9	18.8	23.5	8.2	5.269	< 0.01	2.4	21.2	24.7	35.3	16.5	3.175	< 0.01	< 0.01
	Non-users	1.2	16.5	21.4	44.0	16.9			0.4	10.9	18.8	43.5	26.4			< 0.01
Children who are picky eaters should take dietary supplements to supplement nutrition.	Users	1.2	22.4	16.5	38.8	21.2	2.221	0.03	0.0	10.6	20.0	37.6	31.8	1.316	0.19	< 0.01
	Non-users	0.4	12.4	14.8	44.4	28.0			0.4	8.3	13.2	41.3	36.8			< 0.01
Pregnant women should take dietary supplements to supplement nutrition.	Users	3.6	22.6	27.4	35.7	10.7	1.448	0.15	2.4	15.3	20.0	31.8	30.6	0.606	0.54	< 0.01
	Non-users	2.1	19.8	23.6	38.4	16.1			0.8	11.6	18.3	41.1	28.2			< 0.01
I want to use dietary supplements for weight loss or muscle building.	Users	5.9	25.9	17.7	27.1	23.5	1.157	0.25	5.9	9.4	15.3	43.5	25.9	1.456	0.15	< 0.01
	Non-users	3.3	21.0	16.9	34.6	24.3			2.1	13.7	12.4	34.0	37.8			< 0.01

Users: students who currently use dietary supplements; Non-users: students who previously used or have never used dietary supplements

Statistics analyses were conducted ^a^between users and non-users or ^b^between pre- and post-intervention stages

We also compared pharmaceutical science students with students from other departments (Table 7). Before the intervention, there was a significant difference in the answers to five questions between the pharmaceutical science students and other students. In four out of five questions, the pharmaceutical science students had a better understanding than the other students. These differences remained in the post-intervention phase, even though understanding had generally improved in both groups. On the other hand, on the question “I want to use dietary supplements that have a good reputation”, a greater number of pharmaceutical science students had an incorrect understanding at the pre-intervention stage (z = 2.310, *p* = 0.02). However, this difference disappeared at the post-intervention phase (z = 1.058, *p* = 0.29).

**Table 7:** Comparison of knowledge about dietary supplements between pharmaceutical students and others

		Pre-intervention							Post-intervention							
		Strongly agree (%)	Agree (%)	Neither agree nor disagree (%)	Disagree (%)	Strongly disagree (%)	Z score	*p*-value^a^	Strongly agree (%)	Agree (%)	Neither agree nor disagree (%)	Disagree (%)	Strongly disagree (%)	Z score	*p*-value^a^	*p*-value^b^
Dietary supplements are safe, because they are just food items.	Pharmaceutical	1.6	32.1	30.4	31.0	4.9	−2.072	0.04	1.1	10.4	24.6	49.7	14.2	−2.581	0.01	< 0.01
	Others	4.2	44.4	18.8	29.2	3.5			1.4	22.4	22.4	45.5	8.4			< 0.01
Dietary supplements made from natural ingredients or herbs are safe.	Pharmaceutical	0.6	40.4	32.2	21.9	4.9	−2.456	0.01	1.1	10.4	27.9	49.7	10.9	−3.667	< 0.01	< 0.01
	Others	5.6	48.6	23.6	20.1	2.1			3.5	25.1	28.7	34.3	8.4			< 0.01
Food additives should be avoided.	Pharmaceutical	2.7	35.3	34.2	26.6	1.1	0.243	0.81	6.6	36.6	32.8	21.9	2.2	1.357	0.17	0.03
	Others	4.2	38.2	24.3	26.4	6.9			5.0	34.8	28.4	25.5	6.4			0.66
Dietary supplements made from food items are safe.	Pharmaceutical	1.6	44.0	31.5	21.2	1.6	−0.895	0.37	1.1	12.6	24.0	49.2	13.1	−3.191	< 0.01	< 0.01
	Others	7.0	43.0	26.8	21.1	2.1			0.7	21.7	33.6	35.7	8.4			< 0.01
The efficacy of commercial dietary supplements is confirmed and reliable.	Pharmaceutical	1.6	15.2	36.4	39.7	7.1	−0.757	0.45	1.1	6.0	14.7	51.1	27.2	−2.646	0.01	< 0.01
	Others	2.8	23.1	30.1	32.2	11.9			0.7	14.1	22.5	40.9	21.8			< 0.01
I want to use dietary supplements that have a good reputation.	Pharmaceutical	2.2	38.6	16.9	33.2	9.2	2.310	0.02	1.6	8.2	18.5	47.8	23.9	1.058	0.29	< 0.01
	Others	4.9	22.9	21.5	31.9	18.8			1.4	9.2	17.0	39.7	32.6			< 0.01
Dietary supplements recommended by health professionals are effective.	Pharmaceutical	0.0	17.4	22.8	43.5	16.3	1.481	0.14	0.5	4.9	10.9	53.8	29.9	0.024	0.98	< 0.01
	Others	2.1	14.0	22.4	43.5	29.4			1.4	6.3	15.5	40.9	35.9			< 0.01
Dietary supplements can be used concomitantly with medicines.	Pharmaceutical	0.5	6.0	13.6	42.9	37.0	−3.174	< 0.01	0.5	0.5	6.5	39.7	52.7	−1.654	0.10	< 0.01
	Others	2.1	12.6	16.8	45.5	23.1			0.0	5.6	13.3	32.9	48.3			< 0.01
Dietary supplements can prevent diseases.	Pharmaceutical	0.5	28.3	26.1	34.8	10.3	1.243	0.21	1.1	15.2	14.1	47.8	21.7	−0.228	0.82	< 0.01
	Others	1.4	25.0	20.8	38.2	14.6			2.1	17.6	18.3	33.8	28.2			< 0.01
Dietary supplements can treat diseases.	Pharmaceutical	0.0	6.0	17.4	40.2	36.4	−0.891	0.37	0.0	3.3	6.5	41.3	48.9	−2.011	0.04	< 0.01
	Others	0.7	7.0	19.6	39.9	32.9			0.0	2.8	17.5	38.5	41.3			< 0.01
Dietary supplements can compensate for an unbalanced diet.	Pharmaceutical	0.5	23.9	21.7	40.8	13.0	−0.150	0.88	1.1	12.1	19.8	44.0	23.1	−0.427	0.67	< 0.01
	Others	3.5	24.3	19.4	36.1	16.7			0.7	15.5	21.1	38.0	24.7			< 0.01
Children who are picky eaters should take dietary supplements to supplement nutrition.	Pharmaceutical	1.1	12.0	16.9	47.3	22.8	0.427	0.67	0.5	6.0	10.9	47.8	34.8	−1.445	0.15	< 0.01
	Others	0.0	18.8	13.2	37.5	30.6			0.0	12.6	20.3	30.8	36.4			0.23
Pregnant women should take dietary supplements to supplement nutrition.	Pharmaceutical	1.6	11.5	25.7	46.5	14.8	−3.724	< 0.01	0.6	6.0	14.2	49.2	30.1	−3.676	< 0.01	< 0.01
	Others	3.5	32.2	23.1	26.6	14.7			2.1	21.0	24.5	25.2	27.3			< 0.01
I want to use dietary supplements for weight loss or muscle building.	Pharmaceutical	4.4	22.8	17.9	34.8	20.1	1.330	0.18	3.3	10.3	10.3	41.9	34.2	−0.988	0.32	< 0.01
	Others	3.5	21.5	16.0	29.9	29.2			2.8	15.5	16.9	29.6	35.2			< 0.01

Pharmaceutical: *n* = 184, Others: *n* = 144

Statistics analyses were conducted ^a^between pharmaceutical students and other students or ^b^between pre- and post-intervention stages

## Discussion

We evaluated whether a lecture-based educational intervention could change students’ understanding with respect to the safety and efficacy of dietary supplements. Dietary supplements are frequently misunderstood by college students, even by those who study pharmacology and regularly have lectures about nutrients and dietary supplements. However, these lectures are usually concerned with the law and the regulatory authorities of nutrients and dietary supplements. Conversely, our lecture was comprised of information concerning the safety, quality, risks of interaction with medicines, and the potential adverse events of dietary supplements. In addition, it also addressed some alerts on dietary supplements from Japanese ministries. The results of this study suggest that a once-off educational intervention could improve students’ understandings of dietary supplements.

The prevalence of dietary supplement use shown in this study was higher than in our previous internet survey conducted on college students in Japan [3]. This variation may be due to differences in the participants since the previous study had been conducted on college students that belonged to various departments, whereas more than half of the students in this study belonged to pharmaceutical science departments. It has been reported in other countries that medical/pharmaceutical or health professional students were more likely to use dietary supplements than the general population or other college students [17, 19, 24]. It has also been reported that dietary supplement users believe in the benefits of dietary supplements more than non-users [25]. It is noteworthy that in this study, users of dietary supplements had more positive attitudes towards them than non-users (Table 6). However, the intervention provided a greater degree of improvement in understanding among users than non-users. Therefore, targeted education would appear to be beneficial for users.

Vitamin/mineral supplements may be beneficial for consumers who cannot obtain sufficient nutrients from food items. College students may come to believe that they lack sufficient nutrients from the food items they consume, so they begin to use vitamin/mineral supplements. However, many are unlikely to know which vitamins or minerals are needed. In addition, not only do most dietary supplements provide large amounts of nutrients, but some products also contain greater amounts of nutrients than their labels claim [26]. It has been reported that U.S. dietary supplement users had a higher prevalence of excessive intake of certain vitamins [27]. Habitual dietary supplement use without consulting with health professionals could increase the risk of adverse events due to excessive intake, even if it is only intended to be a vitamin/mineral supplement. In this study, only a few users had obtained information from health professionals such as pharmacists or dietitians, while most obtained information on dietary supplements via the internet, family members, or in-store advertisements. There is a lot of attractive information about dietary supplements on the internet that is not evidence-based [28, 29]. Family members may also get their information via the internet. Similarly, in-store advertisements want to sell their products for profit with little regard for their consumers’ benefit. Education that is focused on the importance of taking adequate amounts of nutrients as part of a balanced diet is needed to prevent unnecessary dietary supplement use among college students.

Consumers have been reported to often misunderstand safety considerations in relation to herbal products [15], with most consumers believing that dietary supplements made from botanical materials are safe because these products claim to be completely natural. The safety of individual ingredients has not always been fully evaluated and some adverse events associated with herbal supplement use have been reported [7, 8, 30]. In addition, many dietary supplements come in the form of pills or capsules, involving not only concentrated active ingredients, but also other possibly harmful or unknown foodstuff ingredients. In this situation, consumers might easily and unwittingly consume an excess amount of ingredients from dietary supplements compared to what they might ingest in their usual diet. Case reports have shown that dietary supplements made from common food items often cause adverse events. For example, green tea is one of the most popular beverages in Japan and is generally consumed safely. However, dietary supplements containing green tea extracts have been reported as a potential cause of liver damage [31, 32]. Educational interventions could help avoid misunderstandings such as “Dietary supplements made from natural ingredients or herbs are safe” and “Dietary supplements made from food items are safe”.

In this study, we conducted an educational intervention on students from four colleges, with more than half of the participants being pharmaceutical science students. The purpose of this study was to evaluate a lecture-based educational intervention on dietary supplements for college students, especially pharmaceutical science students. The concomitant use of dietary supplements and drugs in patients is a social issue not only in Japan but in other countries as well [33, 34]. In this regard, pharmacists play an important role in helping to prevent adverse events. In this study, a greater number of pharmaceutical students had an appropriate understanding of the question “Dietary supplements can be used concomitantly with medicines” compared to other students but some did not. Interventions such as the one in this study could help improve their understanding. This is important because reports have indicated that some pharmacists’ knowledge of dietary supplements remains inadequate, in which case those pharmacists would be less likely to ask their patients about their dietary supplement use [5, 21]. To avoid miscommunication between pharmacists and patients, pharmacists should have appropriate knowledge of dietary supplements. It was for this reason that we conducted an educational intervention involving a significant number of students belonging to pharmaceutical sciences departments who will act as consultants for patients in the near future.

There are some limitations to this study. First, we conducted this study at only four colleges from three types of academic programs in Japan. It is reported that there were 764 colleges and 2307 departments in Japan in 2017. It is not clear whether a lecture-based educational intervention would be effective in other colleges and departments. However, many college students use dietary supplements [3] and receiving appropriate education about them is important. We previously reported that the purpose of using dietary supplements differed between males and females in college students [3]. In this survey, a greater number of males tended to use dietary supplements for athletic purposes than females, but this finding was not statistically significant and may be due to the small sample size in this study. In addition, all of the participating students received the intervention lecture with no control group established to compare with students who did not attend. This would have allowed for a more precise evaluation. However, we did set one control question, namely “Food additives should be avoided,” which was a question not covered in the lecture. There was no difference in understanding between the pre- and post-intervention phases concerning this question. This result suggests that the educational intervention worked well. However, we did not assess the influence of the intervention on student attitudes towards dietary supplements in terms of whether they afterward stopped using dietary supplements or changed their lifestyle habits. In addition, we only assessed changes of understanding concerning dietary supplements by comparing pre- and post-intervention responses to a questionnaire. The long-term effects of providing targeted education remain unclear and this question requires a follow-up study.

## Conclusions

We conducted a single lecture-based educational intervention on college students, involving many from pharmaceutical sciences departments in particular. This study showed that a lecture-based educational intervention about dietary supplements on college students improved their knowledge, despite it only being a one-hour lecture. We consider this type of targeted education to likely be helpful in both avoiding adverse effects among ordinary consumers of dietary supplements as well as better informing future pharmacists in their healthcare relationships with patients. Further studies to evaluate a single lecture-based educational intervention that includes a control group and a consideration of the long-term effects of this type of targeted education is needed.

## Supplementary information

**Additional file 1.**

## Data Availability

All data generated or analyzed during this study are included in the published article.

## Abbreviation

FDA: U.S. Food and Drug Administration
