# Supplementary material for: An educational intervention improved knowledge of dietary supplements in college students
Source: BMC Public Health. 2020 May 7;20:633. doi: 10.1186/s12889-020-08786-3 (PMC7204311; doi:10.1186/s12889-020-08786-3)
Supplement: Supplementary file 1 — Additional file 1. [file 12889_2020_8786_MOESM1_ESM.docx]

Supplemental Table

| Questions | Reasons why "strongly disagree" is the most preferable answer in this study |
| --- | --- |
| Dietary supplements are safe because they are just food items. | Dietary supplements not only contain vitamins and minerals but also contain herbs that have not been confirmed as safe for human consumption. In addition, the quality of some of these products is low. |
| Dietary supplements made from natural ingredients or herbs are safe. | Herbs contain many unknown compounds that may pose health risks. Sometimes, toxic compounds or heavy metals in herbs are condensed in dietary supplements. |
| Food additives should be avoided. | This is a control question. We did not mention food additives in this lecture. |
| Dietary supplements made from food items are safe. | Most food items are safe if they are properly processed. However, food ingredients are extracted and concentrated in dietary supplements. It is easy to take a huge amount of a specific component that cannot be consumed in a regular meal. |
| The efficacy of commercial dietary supplements is confirmed and reliable. | Except for “Foods with health claims” in Japan, the efficacy of commercial dietary supplements are generally confirmed in vitro and in vivo experiments but not in humans. |
| I want to use dietary supplements that have a good reputation. | Good reputations may be the result of effective marketing on behalf of the suppliers. Therefore, they do not promise the efficacy and safety of dietary supplements. |
| Dietary supplements recommended by health professionals are effective. | It has been reported that health professionals do not possess enough knowledge about dietary supplements. In addition, some health professionals have conflicts of interest with manufactures. |
| Dietary supplements can be used concomitantly with medicines. | It is reported that some ingredients in dietary supplements may affect medicinal effects either directly or indirectly. In addition, there is currently not enough information about food-drug interactions in the ingredients of most dietary supplements. |
| Dietary supplements can prevent diseases. | Dietary supplements are not drugs. It is illegal to promote dietary supplements for disease prevention in Japan. |
| Dietary supplements can treat diseases. | Dietary supplements are not drugs. It is illegal to promote dietary supplements for disease treatment in Japan. |
| Dietary supplements can compensate for an unbalanced diet. | Some dietary supplements contain vitamins and minerals in a good balance but most of them do not. Some of them contains inordinate amounts of these vitamins or minerals. |
| Children who are picky eaters should take dietary supplements to supplement nutrition. | In some cases, it is helpful for picky eaters to consume vitamins and minerals. However, most dietary supplements are not confirmed to be safe for children. |
| Pregnant women should take dietary supplements to supplement nutrition. | The Japanese government recommends pregnant women to take 400 μg folic acid/day. However, most folic acid supplements contain not only folic acid but also other ingredients/herbs that have not been safely confirmed in pregnant women. |
| I want to use dietary supplements for weight loss or muscle building. | There have been many reports that consumers experienced adverse events associated with the use of dietary supplements for weight loss or muscle building not only in Japan but also in other countries. |
